# Functional outcomes and complications of hook plate for bony mallet finger: a retrospective case series study

**DOI:** 10.1186/s12891-021-04163-2

**Published:** 2021-03-16

**Authors:** Wei-Chih Wang, Cheng-En Hsu, Chen-Wei Yeh, Tsung-Yu Ho, Yung-Cheng Chiu

**Affiliations:** 1 School of Medicine, China Medical University, Taichung, Taiwan; 2 Department of Orthopedic Surgery, China Medical University Hospital, No. 2, Yude Road, North District, Taichung City, Taiwan 404472 Taichung, Taiwan; 3 Department of Orthopedics, Taichung Veterans General Hospital, Taichung, Taiwan; 4 Sports Recreation and Health Management Continuing Studies-Bachelor’s Degree Completion Program, Tunghai University, Taichung, 407 Taiwan

**Keywords:** Mallet fracture, Bony mallet finger, Open reduction and internal fixation, Implant problems, Nail bed deformity, Screw loosening

## Abstract

**Background:**

The treatment of mallet fracture using hook plate fixation was first introduced in 2007 and has subsequently shown excellent outcomes. Common complications, such as nail deformity and screw loosening, have also been reported. Very few studies have focused on these common complications or their prevention. In this study, we present the clinical outcomes and complications of our case series and describe the pitfalls and detailed solution of surgical tips to avoid common complications related to this procedure.

**Methods:**

The retrospective case series of 16 patients with mallet fractures who underwent open reduction and hook plate fixation in our hospital from 2015 to 2020 were retrospectively reviewed. Data on extension lag, range-of-motion (ROM) of the distal interphalangeal joint (DIP) joint, the Disabilities of the Arm, Shoulder, and Hand (DASH) score, and surgical complications were collected and analysed. The clinical outcome was graded according to the Crawford mallet finger criteria.

**Results:**

Sixteen patients were included in our analysis. The median DIP extension lag was 0° (range, 0° to 30°) and the median active DIP flexion angle was 60° (range, 40° to 90°). The median DASH score was 0 (range, 0–11.3). Fourteen patients with good and excellent results were satisfied with this treatment. The Complication rate in our patient series was 18%. Common complications reported in articles included wound necrosis, extension lag, nail deformity, and plate loosening.

**Conclusions:**

Despite the fact that the treatment of mallet fracture with hook plate fixation has satisfactory functional outcomes, pitfalls, including iatrogenic nail germinal matrix injury, unnecessary soft tissue dissection, and insufficient screw purchase, were still reported. To avoid complications, we suggest modifications of the skin incision, soft tissue dissection, and screw position.

## Background

Mallet finger injuries are usually caused by excessive flexion force on the distal interphalangeal (DIP) joint. They can be divided into two types: Tendinous mallet finger is a rupture of the extensor tendon in Zone 1, and bony mallet finger is an avulsion fracture of the extensor tendon from the distal phalangeal base. Mallet finger leads to extensor mechanism imbalance between the proximal interphalangeal (PIP) and DIP joints, which may give rise to a swan neck deformity if left untreated. The majority of mallet finger injuries can be treated non-surgically, but surgical intervention is required in circumstances where the fracture fragment involves more than one-third of the articular surface, in the event of fracture dislocation, and for people who are unable to protect exposed pins and who cannot tolerate a period of immobilisation [1].

Various surgical techniques have been reported for mallet fracture fixation, including DIP joint pinning, dorsal blocking pin, tension band wire, and hook plate fixation [2–6]. However, the main difficulty with these surgical techniques including fragment splitting and suboptimal reduction, and potential complications of functional limitation, synarthrosis, pin tract infection, skin necrosis, nail deformity, and residual pain. The accumulated incidence rate of the above complications was up to 47% [4]. Open reduction with hook plate fixation allows direct fracture reduction and avoids pin exposure, which is clearly advantageous in terms of postoperative care. The technique was first introduced by L. C. Teoh [6]; subsequently, good to excellent surgical outcomes have been reported by many authors, although surgical complications, including recurrence of joint subluxation, screw loosening, skin necrosis, and nail deformity, have also been reported, with incidence rates ranging from 0 to 23% [6–13]. Despite the relatively high complication rates, there is little discussion in the literature regarding surgical techniques that can be employed to avoid these complications. Therefore, the purpose of this study was to report our clinical outcomes and to share surgical tips that we have found greatly reduce the risk of complications in the treatment of mallet fracture with hook plate fixation.

## Methods

### Patient enrolment

This study protocol was approved by a local research ethics committee (IRB number CMUH109-REC1–058), all methods were carried out in accordance with relevant guidelines and regulations. Between January 2015 and July 2020, we retrospectively reviewed 20 patients who underwent hook plate fixation in our hospital. All surgeries were performed by a senior hand surgeon (Dr. Y.C.Chiu). All 20 mallet fractures were closed fractures. Four patients were excluded because the follow-up duration was less than 12 months. Sixteen patients were included in the final analysis.

### Surgical indications

This procedure was indicated in selective patients: those with intra-articular avulsion fracture with fragments involving more than 30% of the joint surface of the distal phalanx, or for patients who were unable to protect exposed pins or could not tolerate a period of immobilisation for the requirement of work. The reason why we chose fracture fragment> 1/3 articular surface as our surgical indication was as follows:1. When fracture fragment> 1/3 articular surface, intramedullary DIP joint pinning as common alternative treatment has a high risk to hinder fracture reduction 2. Larger fragment provides a stronger base for the hook plate to hook on, avoids iatrogenic fracture and tendon detachment.

All surgeries were performed within 2 weeks of the injury. Exclusion criteria were tendinous mallet finger, injury which happened more than 2 weeks and follow-up time less than 12 months.

### Operative methods

**Fig. 1:**
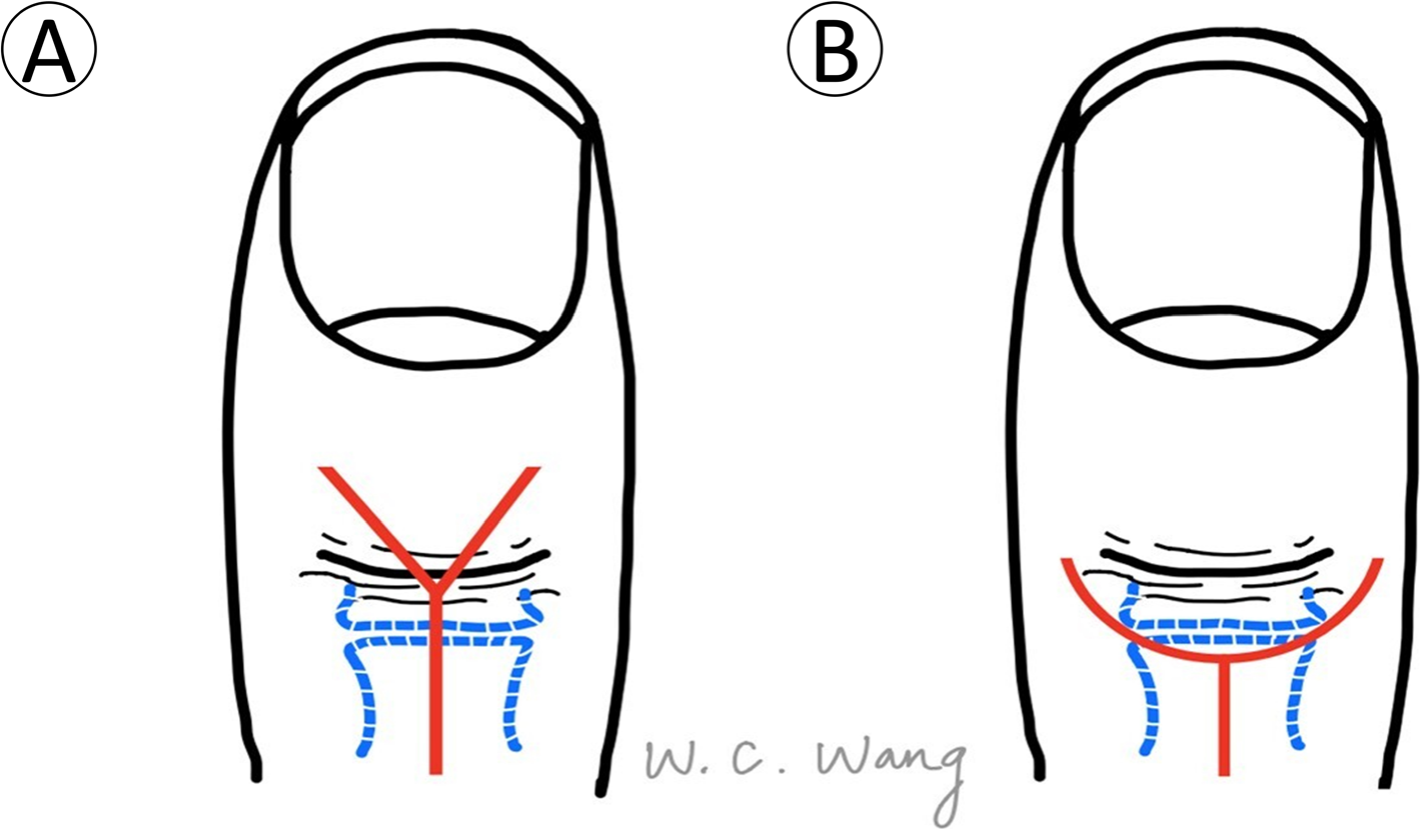
Modified skin incision. **a** Traditional Y incision (**b**) Modified Y incision (Lazy Y incision). Compared to the typical Y incision, the wider distal-based flap made by the modified Y incision better preserved blood supply from the digital arteries. The centre of the Y incision was positioned 0.5 cm proximal to the DIP joint

**Fig. 2:**
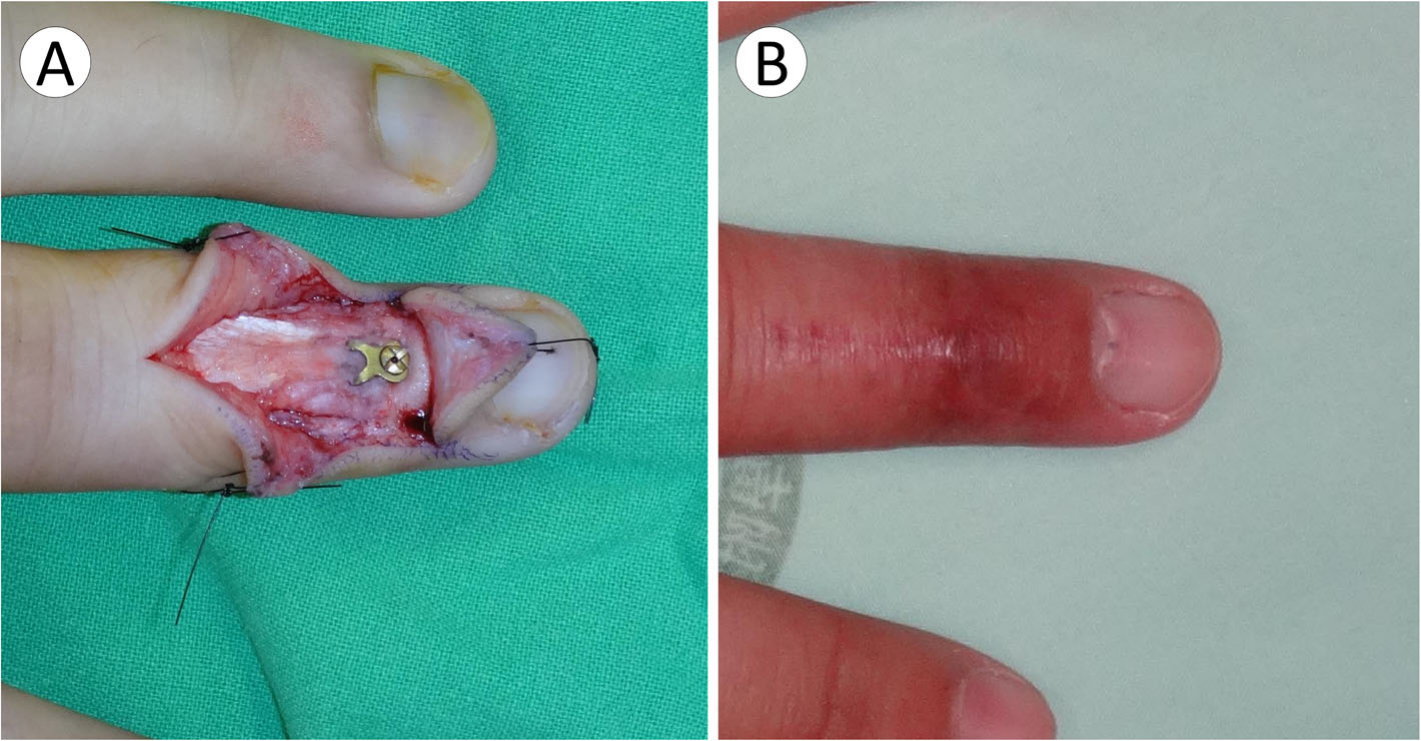
Lazy Y incision and hook plate fixation. **a** Lazy Y incision and hook plate were applied on the DIP joint. **b** Good wound healing was shown at 1-month follow-up

The injured hand was prepared using a standard sterile procedure. A digital nerve block with lidocaine (1% lidocaine) was used, and haemostasis was achieved using a glove tourniquet. A Lazy-Y incision was made 0.5 cm proximal to the main DIP crease (Figs. 1 and 2). The wide-based skin flap was meticulously dissected from the extensor tendon. The bony fragment was identified and confirmed using a C-arm image intensifier. The integrity of the terminal tendon-to-bone structure was carefully preserved. We found this to be the critical step in preventing postoperative extension lag of the DIP joint. Meticulous care should also be taken not to damage the germinal matrix when the skin flap is dissected distally. The germinal matrix was subperiosteally elevated 3–4 mm in preparation for the implant placement. We found this to be the critical step in preventing nail deformity.

**Fig. 3:**
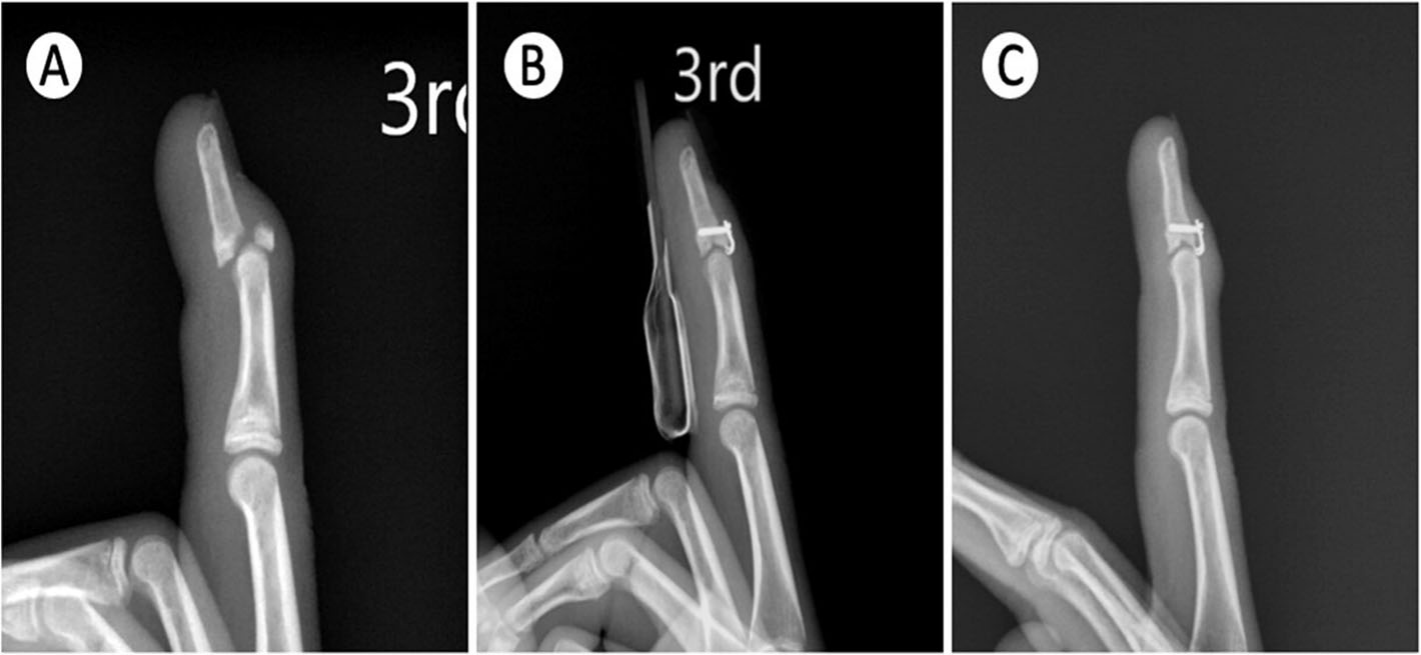
Union of mallet fracture. **a** The pre-operative radiograph showed mallet fracture with DIP joint subluxation. **b** The post-operative radiograph showed that the fracture was reduced and fixed with the hook plate. **c** Bone healing was shown on the 3-month follow-up radiograph

Subsequently, a 1.7-mm non-locking hook plate (Stryker hand plating system) was applied proximally to the terminal tendon-to-bone junction and distally under an elevated germinal matrix. Reduction of the avulsed fracture was temporarily achieved by holding the hook plate to the distal phalanx with a Kelly clamp. The hook should precisely catch the lip of the joint surface with no interference from the DIP joint (Fig. 3) and the fracture gap should be less than 1 mm. Full extension of the DIP joint was temporarily held by a 1.0 Kirschner wire. A pilot 1.0 Kirschner wire was then drilled into the screw hole to ensure the exact screw axis. The screw axis should be aligned obliquely toward the fingertip to increase the bony purchase length. An intraoperative C-arm image intensifier was used to check the hook plate position, pilot Kirschner wire trajectory, and fracture reduction. The pilot 1.0 Kirschner wire was then removed. The screw hole was drilled along the tract of the pilot 1.0 Kirschner wire and a 1.7-mm screw was inserted to fix the hook plate on the distal phalangeal bone. The screw should be purchased through the near and far cortex to ensure adequate fixation force and fracture stability for early rehabilitation. Typically, the length of the screw was approximately 6 mm. Surgeons should be cautious not to insert the screw into the fracture gap, which may cause early failure of fixation. After screw fixation and adequate irrigation, the wound was closed using 5–0 non-absorbable sutures.

### Postoperative course and follow-up

All patients received wound care and prophylactic IV antibiotics for one dose. They were discharged from the hospital the day after surgery. Postoperative finger splint protection was maintained for 2 weeks until the first visit to remove stitches. The rehabilitation program, which included full active and passive motion of the DIP joint, was initiated 2 weeks postoperatively. Patients were followed up monthly until bone union was observed.

### Treatment outcomes assessment

Clinical outcome assessments included active range-of-motion of the DIP joint, angle of extension lag, Dash score and visual analogue scale (VAS) pain score. The clinical outcome was graded according to the Crawford mallet finger criteria [14].

Radiographic images were arranged at 4-week intervals to assess fracture union. The imaging studies were analysed in detail to evaluate complications, including bony conditions, such as malunion, plate loosening, and soft tissue conditions, such as skin necrosis and nail bed deformity. All imaging analysis were performed by the treating surgeon who had experience in hand surgery more than 10 years.

## Results

Twenty patients who were treated with hook plate and 16 patients were included in the final analysis (Table 1). The median age was 31 years (range, 18–51 years). The injury mechanisms were sports injury, occupational accidents, and traffic accidents. Seven patients were female and nine were male. All fractures were classified using the Wehbe and Schnieder classification, that is, based on the degree of joint subluxation and the size of the avulsion fragment [15] (Table 2).

**Table 1:** Demographics of 16 patients

Patient No.	Age	Gender	Hand L / R	Digit	Mechanism of injury	Wehbe and Schneider’s classification	Injury to surgery (days)	Follow-up (Months)
1	23	F	R	4	Basketball	IB	12	35
2	31	M	L	3	Basketball	IB	8	32
3	39	F	L	5	Work	IB	7	30
4	19	M	R	3	Basketball	IC	5	33
5	22	F	R	4	Basketball	IB	5	29
6	34	M	L	3	Basketball	IB	5	32
7	31	M	L	4	Traffic accident	IB	12	28
8	18	M	R	3	Basketball	IIB	6	26
9	40	M	R	5	Work	IIC	1	19
10	42	M	L	5	Work	IIC	8	14
11	33	F	R	3	Traffic accident	IB	3	12
12	23	F	R	4	Sport	IB	1	15
13	42	F	R	3	Fall	IIB	3	14
14	23	M	R	5	Work	IIC	2	12
15	51	F	L	5	Work	IC	3	13
16	28	M	L	3	Basketball	IB	5	12

**Table 2:** Wehbe and Schneider classification [15]

Type	1	No DIP joint subluxation
	2	DIP joint subluxation
	3	Epiphyseal and Physeal injury
Subtype	A	< 1/3 of articular surface involvement
	B	1/3 to 2/3 articular surface involvement
	C	> 2/3 articular surface involvement

Among the 16 mallet fractures, nine fractures with 1/3–2/3 articular involvement were classified as type 1B, two fractures with more than 2/3 articular involvement were classified as 1C; five fractures with DIP joint subluxation were classified as IIB and IIC.

The median interval from injury to surgery was 6 days (range, 1–12 days). The median number of weeks required for union according to radiographic results was 8 (range, 6–10) weeks (Table 3). The median number of follow-up months was 26 (range, 12–35) months (Table 1).

**Table 3:** Surgical outcomes for 16 mallet fractures treated with hook plates

Patient No	DIPJ Extension lag	DIPJ Flexion	DIPJ ROM	Crawford Criteria	Dash Score	VAS	Complication	Union time (weeks)	Implant Removal
1	10	70	60	Good	5	0	No	7	Nil
2	0	55	55	Excellent	0	0	No	6	Nil
3	25	90	65	Fair	6.3	2	No	8	Nil
4	0	50	60	Excellent	0	0	**Nail deformity**	8	Nil
5	5	50	45	Good	0	0	**Nail deformity**	9	**Yes**
6	0	60	60	Excellent	11.3	0	No	6	Nil
7	10	50	40	Good	0	0	No	9	Nil
8	0	80	80	Excellent	0	0	No	8	Nil
9	30	70	40	Poor	0	0	**Loss of reduction**	10	**Yes**
10	10	90	80	Good	0	0	No	7	Nil
11	0	70	70	Excellent	0	0	No	6	Nil
12	0	60	60	Excellent	0	0	No	7	Nil
13	0	60	60	Excellent	0	0	No	7	Nil
14	5	70	65	Good	5	0	No	6	Nil
15	0	60	60	Excellent	0	0	No	8	Nil
16	0	70	70	Excellent	0	0	No	7	Nil

The detailed clinical outcome is demonstrated in Table 3. The median DIP extension lag was 0° (range, 0° to 30°). The median flexion was 60° (range, 40° to 90^o^). The median DASH score was 0 (range, 0–11.3). Patient 3 had residual pain at the time of the last follow-up (VAS score 2). According to the Crawford criteria, 9 digits (57%) achieved an excellent result, 5 digits (31%) a good result, 1 digit (6%) a fair result and 1 digit (6%) a poor result (Table 4). Fourteen patients (88%) were satisfied with this treatment.

**Table 4:** Crawford criteria [14]

Crawford outcome	DIP extension lag	DIP flexion	Pain
Excellent	0 degree	Full	None
Good	1–10 degree	Full	None
Fair	11–25 degree	Any loss	None
Poor	> 25 degree	Any loss	Residual pain

**Fig. 4:**
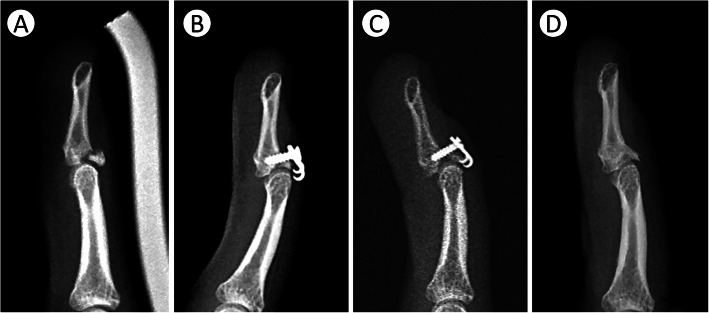
Suboptimal plate and screw placement. **a** The pre-operative radiograph showed mallet fracture without DIP joint subluxation. **b** The post-operative radiograph showed that the screw was inserted through the fracture site and failed to purchase the far cortex. **c** Implant loosening with fracture fragment displacement was found at 1-month follow-up. **d** The hook plate was removed after bone union

The overall complications rate was 18% (3 of 16 patients), including two patients with nail deformity (12%) and one patient with implant loosening (6%) (Table 3). For patients with nail deformity, one patient recovered after removal of the implant and one declined implant removal operation. Implant loosening was observed in one patient. Radiographs at the 2-month follow-up visit showed loss of reduction and screw loosening (Fig. 4a-c). The implant was removed after bone union was observed (Fig. 4d); however, an extension lag of 30° persisted.

## Discussion

Teoh and Lee described an innovative technique to manage bony mallet fracture with a hook plate, with nine of nine patients (100%) showing a good to excellent Crawford outcome [6, 14]. All patients were satisfied with the postoperative ROM of the DIP joint. They reported nearly normal flexion motion without an extension lag. However, nail deformity was discovered in three of nine cases. With the popular application of the hook plate, later studies showed an increased prevalence of related complications, such as plate extrusion, skin necrosis of the operation site, and screw loosening (Table 5). The incidence of nail deformity in the literature ranges from 0 to 18%, which is similar to the rate found in our study [5–11, 16, 17]. In our case series, 14 of 16 patients with mallet fracture were satisfied with the hook plate treatment. The median extension lag and DASH score were 0° and 0, respectively. The incidence of nail deformity and screw loosening was 12 and 6%, respectively. No skin necrosis was noted in our patients.

**Table 5:** Clinical studies evaluating outcome and complications of hook plate fixation for mallet fracture

Authors	Number of patients	Surgical indication	Results						
			Mean follow up	Mean DIP Flexion	Mean DIP extension loss	Crawford Criteria EGFP^a^ %	Other evaluation methods	Complications	Implant removal
Teoh and Lee (2007) [6]	9	Fractures > 1/3 articular surface w/ subluxation distal phalanx	17 (6–37) months	64^o^ (range 60^o^–70^o^)	0^o^	E44, G56%	Warren and Norris 100% success	3 nail deformity	0
Kanthan Theivendran et al. (2007) [5]	1	DIP jpint avulsion fracture w/ volar subluxation	6 months	70^o^	8^o^	G:100%	DASH: 0 point	0	0
Szalay et al. (2011) [7]	59	Doyle IVb with displacement > 2 mm	38.3 (3–69) months	77^o^	7^o^	^b^	very good in 85%, good in 9%, and bad in 7%	7 nail deformity; 2 skin perforations	14
Serdar Toker Et al (2015) [8]	6	> 25% articular Surface	12.7 (3–26)months	80^o^	5^o^	E:33% G:33% F:33%	VAS 7.5 ^b^ > 2.0	1 partial skin necrosis and delayed wound healing	0
M. A. Acar1 Et al (2015) [9]	13	> 1/3 articular surface	> 12 months.	64^o^	3^o^	E:61.5% G:38.5%	VAS 0	3 nail deformity	0
Fábio Sano Imoto (2016) [16]	25	> 1/3 articular surface or volar subluxation	18 months	4 pt’(16%): limited flexion	7 pt’ (28%): limited extension	E:40% G:60%	84%: no pain 16%: symptoms mild intensity.	0	0
Joyce Tie (2017) [10]	31	DIPJ subluxation > 30% fragment	8 ± 6 months	56^o^(range 30^o^–80^o^)	Pre-op 22^o^(range 0^o^–50^o^) Post-op 6^o^(range 0^o^–45^o^)	E: 29% G: 26% F: 39% P: 6%	^b^	3 nail deformity; 3 fracture displacement; 3 skin flap ischaemia	22 (71%)
Andal Thirumalai (2017) [11]	35	> 1/3 articular surface	24 months	40^o^	5 ^o^ (F/U in 10 cases)	^b^	^b^	6 Nail deformity 5 Plate extrusion	14 (40%)
H. Vester (2018) [17]	38	> 1/2 articular surface with joint subluxation	> 12 months	^b^	^b^	^b^	SF-36: 50 points^c^	^b^	14 (37%)

^a^Crawford Criteria EGFP, *E* Excellent, *G* Good, *F* Fair, *P* Poor

^b^ Not available

^c^50 points is defined as the baseline of the mean score of predetermined population

Two of the 16 patients with mallet fracture underwent implant removal in this study. One patient complained of nail deformity, and the plate was removed 6 months after the operation. Implant loosening occurred in the other patient, and the plate was removed at 3 months after bone union. A review of the literature (Table 5) revealed that removal of the plate was performed in 14–71% of patients after hook plate insertion. The indication for hook plate removal is usually soft tissue complications [10, 11, 18]. Szalay removed plates from two of 14 (14%) patients who had skin irritation [18]. Tie removed plates from 22 of 31 (71%) patients. Among them, three had plate loosening and three had skin ischaemia [10]. Thirumalai removed plates from 14 of 35 (40%) patients due to skin irritation or nail deformity [11]. In our study, the indications for plate removal were nail deformity and plate loosening. The rate of implant removal (12%) in this series was similar to that reported by other authors. In our experience, removal of the implant is not necessary in most patients without symptoms.

A literature review regarding the related complications after hook plate fixation is shown in Table 5. Table 6 shows several operative techniques that can be used to overcome the pitfalls based on our experience.

**Table 6:** Potential problem after hook plate fixation

Potential problem.	Solutions
Operative wound skin necrosis	**Incision**-Lazy Y skin incision was made to gather more blood supply to skin flap.
Nail deformity	**Germinal matrix protection**-Subperiosteal elevation of germinal matrix about 3-4 mm and put the plate under it.
Terminal tendon laxity with extension lag of DIP joint	**Respect the soft tissue**- Meticulously dissection without injury of the integrity of termial tendon-to-bone structure.
Implant loss reduction (screw loosening)	**Adequate screw purchase**-Oblique trajectory of screw placement with bicortical screw purchase
	**Wide awake test**-Intraoperative wide awake test to ensure adequate strength of fixation. Add Kirschner wire fixation, if wide awake test fail

### Pitfall #1: operative wound skin necrosis

Several surgical approaches have been described for the skin incision. Teoh and Lee used a curved transverse dorsal incision and reported that none of the nine patients developed soft tissue complications [6]. However, some authors have reported that the surgical field was limited when using a linear incision. Tie applied a hook plate using an H-type skin incision; however, three of 29 patients developed transient skin flap ischaemia [10]. Szalay used a typical Y incision, which resulted in two cases of skin perforation in 59 patients [18]. Based on the collective experience described above, we used a Lazy Y incision to improve the surgical field and preserve more blood supply to the skin flap via a wide skin flap base. None of our cases developed skin flap congestion, necrosis, or implant perforation due to poor healing.

### Pitfall #2: nail deformity

**Fig. 5:**
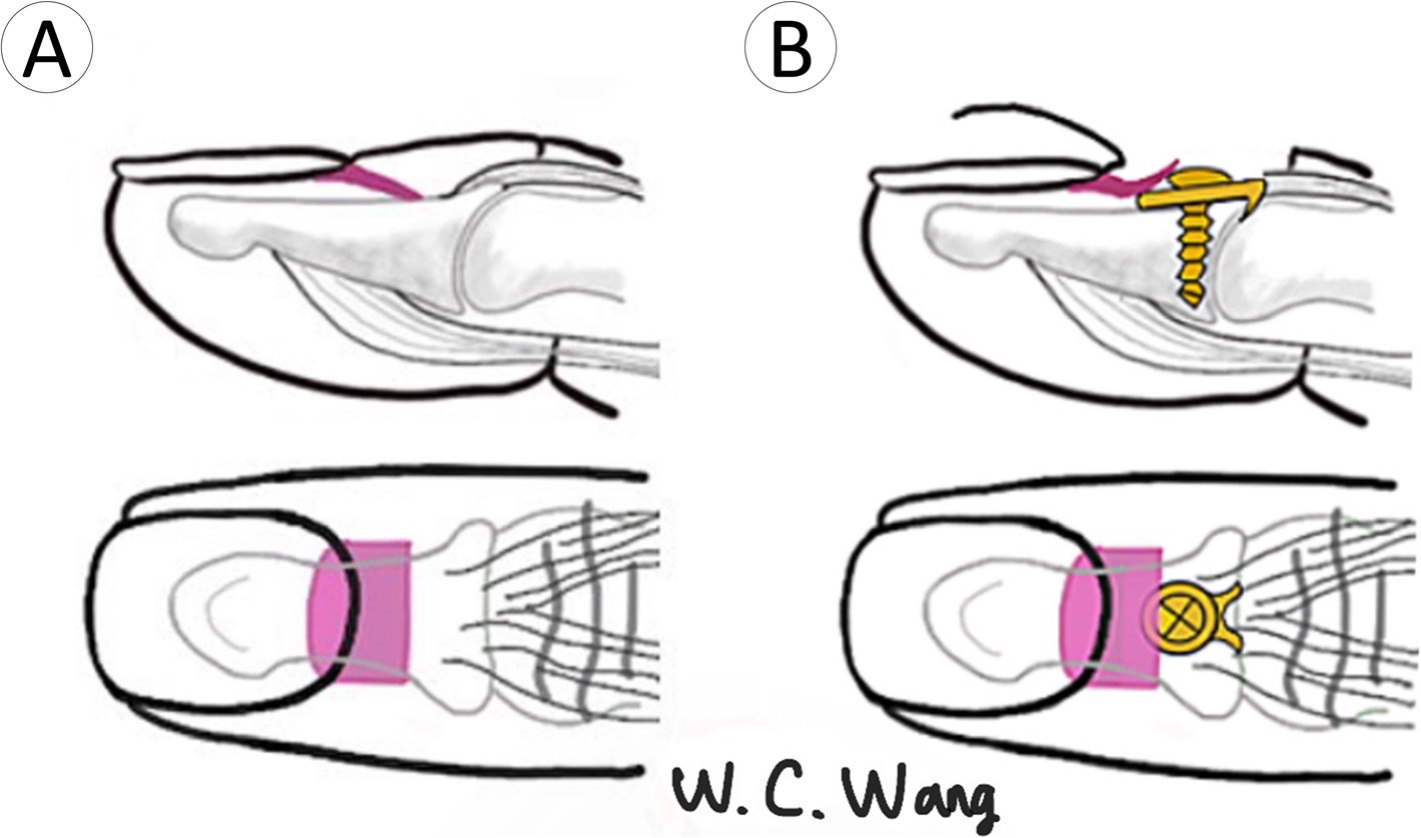
Subperiosteal elevation of germinal matrix. **a** Anatomy of the germinal matrix of the nail bed (purple). **b** Operative method: Plate positioning following 3–4 mm subperiosteal elevation of the proximal germinal matrix

**Fig. 6:**
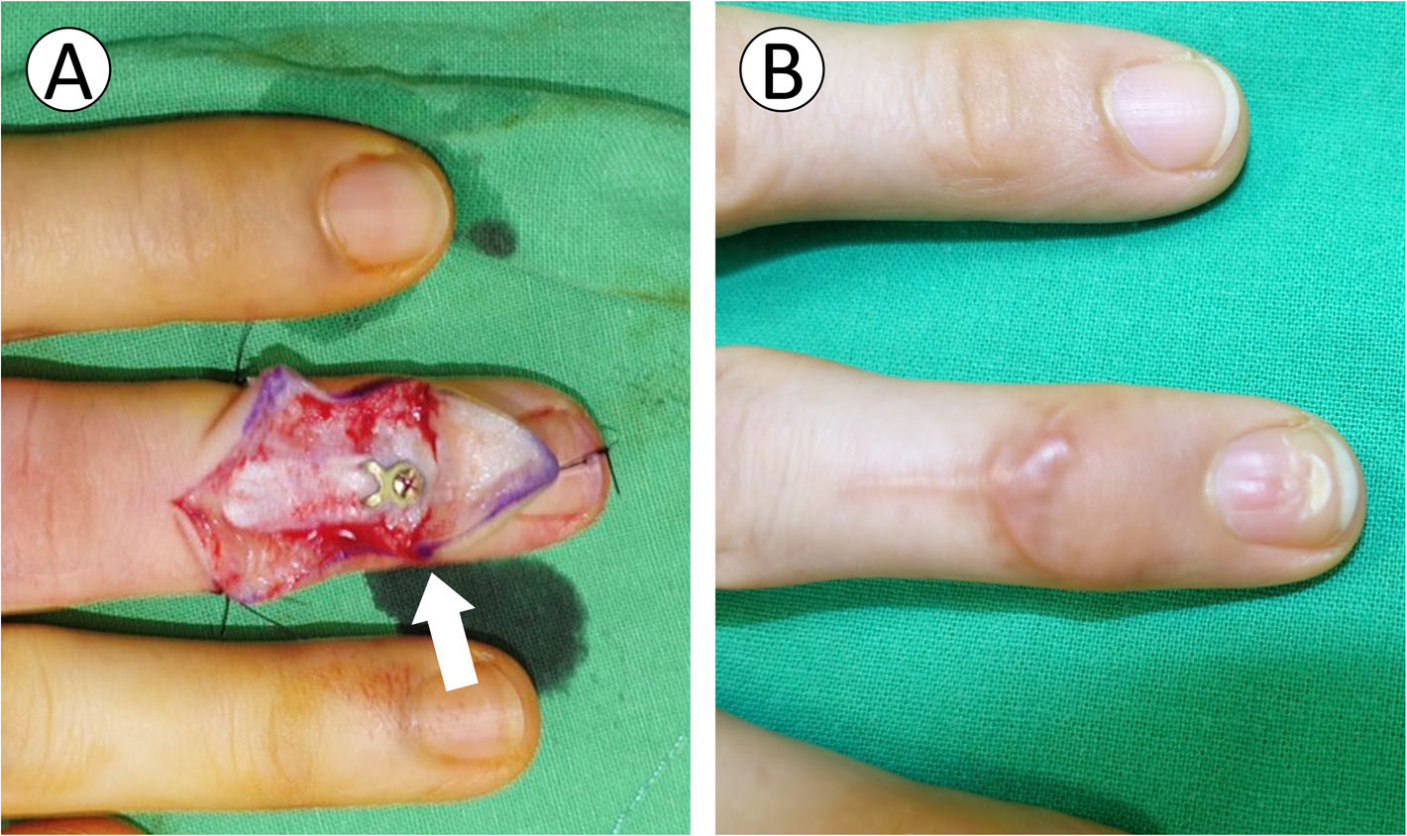
Nail deformity. **a** In case 4, the hook plate was placed directly onto the proximal germinal matrix (white arrow). **b** Severe nail deformity was noted postoperatively at the 4-month follow-up

The main reason for nail deformity was iatrogenic germinal matrix injury due to extensive exposure of the soft tissue during surgery. Schweitzer collected 56 cadaveric digits and investigated the anatomy of the terminal tendon [19]. He found that the average distance between the distal end of the terminal tendon and the proximal edge of the germinal matrix was 1.4 mm (range, 0.9–2.0 mm). The average length of the terminal tendon was 10.1 mm (range, 5.1–15.9 mm). The insertion length of the terminal tendon was 1.2 mm (range, 0.8–1.7 mm) when extended distally from the articular surface. To apply the 6-mm hook plate on the distal phalanx, the germinal matrix must be elevated 3–4 mm periosteally in preparation for implant placement (Fig. 5). Reardon demonstrated the anatomy of the germinal matrix of the nail bed [20]. The length of the germinal matrix was approximately 0.55 times the distance from the proximal nail fold to the DIPJ. The total length of the germinal matrix was approximately 7–8 mm. In our early practice, we attempted not to elevate the germinal matrix and directly placed the plate onto it (Fig. 6a); consequently, cases 4 and 5 were complicated by nail deformity (Fig. 6b). Our current modification of the surgical technique involved a 3–4 mm subperiosteal elevation of the germinal matrix with the plate placed under it. Two common techniques to prevent nail deformity in a previous report included direct germinal matrix incision [10] and subperiosteal elevation of the proximal germinal matrix [5, 6]. There is no consensus regarding which method has a lower complication rate of nail deformity; however, no further nail deformities occurred later in our case series with this modification of surgical technique (subperiosteal elevation of the proximal germinal matrix). Although the exact reason for nail deformity remains unclear, we still recommend a limited subperiosteal dissection approach to preserve the integrity of the germinal matrix during the operation based on our experience.

### Pitfall #3: terminal tendon laxity with post-operative extension lag

The postoperative DIP joint range of motion in our study was compatible with other reports (Table 5). Our median DIP flexion range-of-motion was 60^o^, compared with ROMs of 40^o^ to 80^o^ in eight other studies. The median DIP extension loss in our study was 0^o^ (range, 0° to 30°), which was also consistent with values reported in other studies. Two patients (cases 3 and 9) had an extension lag of more than 10°. In case 9, the extension lag was due to screw loosening and loss of reduction (Fig. 4). In case 3, the extension lag was due to over-dissection of the terminal tendon-to-bone structure. In our earlier cases, we elevated the whole terminal tendon to expose the joint surface for the purpose of achieving excellent fracture reduction. Exposing the joint surface provides a clearer operative field and permits fracture reduction under direct vision, but dissecting the entire tendon-to-bone structure also results in tendon laxity and subsequent extension lag of the DIP joint. After case 3, we switched to meticulous dissection of the soft tissue surrounding the terminal tendon. The integrity of the terminal tendon-to-bone structure was carefully preserved. Without detaching the fracture fragment from the distal phalanx, we placed the hook plate directly overlying the terminal tendon and checked the position of the plate under the C-arm image intensifier. We found that this is a critical step in the prevention of postoperative extension lag.

### Pitfall #4: loss of reduction

**Fig. 7:**
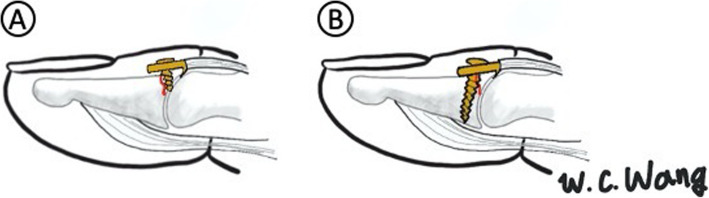
Oblique screw trajectory. **a** For larger fragment, the screw should not be routinely inserted perpendicular to the long axis of the hook plate. **b** The screw was inserted distally to achieve more bony purchase

Loss of reduction was found in one case (case 9) of bony mallet fracture with DIP joint subluxation and more than 60% joint surface involvement. The immediate postoperative radiograph showed a suboptimal position of the plate, and the screw was inserted through the fracture site and failed to purchase the far cortex (Fig. 4b). The radiograph at the 2-month follow-up visit showed screw loosening and loss of reduction (Fig. 4c). In our opinion, hook plate loosening can be avoided by performing surgery in accordance with the following surgical tips. First, when an injured digit has extensive joint surface involvement (over 40%), the screw may not achieve adequate bony purchase when it is inserted perpendicularly to the long axis of the hook plate (Fig. 7). Therefore, the drilling trajectory should be aligned obliquely toward the fingertip. This allows the insertion of a longer screw, which provides greater power for bony purchase. Owing to the fact that there is only one chance to drill the screw hole in such a small phalangeal bone and covered soft tissue may make it difficult to find the optimal screw axis, we suggest drilling a pilot 1.0 Kirschner wire through the screw hole and ensuring the exact screw axis under C-arm image confirmation before definite screw insertion. Second, the strength of the bicortical screw purchase provides better stability than unicortical screw purchase. A screw with a short length (less than 5 mm) may not be sufficient to resist the pulling force of the extensor tendon and may fail when patients begin early motion of the DIP joint. Third, if there is a possibility that the strength of plate and screw fixation may be unsatisfactory, we recommend the addition of extra pin fixation to increase the fixation power, especially under two circumstances: 1. fracture fragment with > 40% articular surface involvement and 2. loss of reduction in the intraoperative active ROM test under wide-awake anaesthesia. The Kirschner wire can be removed 4 weeks after evidence of bone union on radiography.

There were several limitations in this study, including the small number of patients, the retrospective nature of the study, and the lack of preoperative functional outcome measures. Besides, not all patients were treated with exactly the same operative technique. We did modify the surgical technique due to complications in earlier cases. Further studies with a larger population should be conducted to confirm these results.

## Conclusion

The treatment of mallet fractures with hook plates has acceptable functional outcomes and complication rates. It provides anatomic reduction, rigid fixation, and the opportunity for early finger motion, which cannot be achieved with other treatments. Our experience has enabled us to figure out the tips, tricks, and techniques to avoid pitfalls. Common complications, such as surgical skin necrosis, nail bed deformity, and plate loosening, can be avoided through modifications of skin incision, soft tissue dissection, and screw position.

## Data Availability

The datasets used and analyzed during the current study are available from the corresponding author on reasonable request.

## Abbreviations

ROM: Range-of-motion
DIP: Distal interphalangeal joint
DASH: Disabilities of the arm, shoulder, and hand
PIP: Proximal interphalangeal
VAS: Visual analogue scale
